# Assessment of the Hormetic Effect of Arsenic on Growth and Physiology of Two Cultivars of Maize (*Zea mays* L.)

**DOI:** 10.3390/plants11243433

**Published:** 2022-12-08

**Authors:** Beáta Piršelová, Ľudmila Galuščáková, Libuša Lengyelová, Veronika Kubová, Vilma Jandová, Jitka Hegrová

**Affiliations:** 1 Department of Botany and Genetics, Faculty of Natural Sciences and Informatics, Constantine the Philosopher University in Nitra, Nábrežie mládeže 91, 949 74 Nitra, Slovakia; 2 Transport Research Centre, Líšeňská 33a, 636 00 Brno, Czech Republic

**Keywords:** arsenic, maize, growth, photosynthesis, stomata, water balance

## Abstract

Although growth stimulation at low arsenic doses was observed in several plants, few studies have focused on this phenomenon in more detail. The effects of different concentrations of arsenic (0–50 mg kg^−1^ of soil: As0–As50) on the growth and selected physiological parameters of two maize cultivars (*Zea mays* L. cvs. Chapalu and MvNK 333) were tested. Cultivar MvNK 333 manifested a generally higher tolerance to As than cv. Chapalu, which may be related to the lower content of As in the tissues. The highest stimulatory effect of As was recorded at doses of As1 and As2 (cv. Chapalu), and at the As5 dose (MvNK 333), there was an increase in shoot elongation, biomass, and relative water content (RWC), as well as the content of photosynthetic pigments. The stimulatory effect of lower doses of As apparently represents an adaptation mechanism that is associated with water content regulation in the given conditions. The stomata of the studied cultivars were involved in this regulation in different ways. While cv. Chapalu exhibited increased numbers of stomata on both sides of leaves, cv. MvNK 333 instead responded to the given conditions with decreased stomata size. Although hormetic manifestations closely related to changes in stomatal number and size were observed, a typical stomatal hormetic response was not observed in the given range of As doses.

## 1. Introduction

Arsenic (As) is a non-essential and generally toxic element to plants. The worldwide average concentration of As in soil is set at 10 mg kg^−1^, and the maximum permissible limit for agricultural soil recommended by the European Union is 20 mg kg^−1^ [1]. Crops and vegetables cultivated in arsenic-contaminated soil accumulate a significant amount of arsenic, which ultimately enters the human food chain [2]. Arsenic exists in the environment in various organic and inorganic compounds, and the most important inorganic compounds are arsenate As(V) and arsenite As(III) [3]. In aerobic soils arsenates (AsV) dominate, which are less toxic forms of As. Due to their stronger sorption to minerals, they are also less mobile than As(III) compounds [4]. The mechanisms of arsenic toxicity differ widely among chemical species. The chemical similarity of P(V) and As(V) conditions their common transport pathway in plants, because P(V) transporters, despite their higher affinity to phosphorus, are responsible for As(V) uptake. Inside the plant, inorganic As(V) compounds are involved in phosphate metabolism, resulting in the inhibition of oxidative phosphorylation. Unlike As(V), As(III) is transported into plants by aquaporins, specifically by a group of transporters on a cytoplasmic membrane designated as NIP (“Noduline 26-like Intrinsic Protein”) [5]. Arsenic has been determined mainly to be As(III) in various plant parts. The high prevalence of As(III) in plants results from the immediate reduction of As(V) received by the plant to As(III). The ACR2 gene encoding arsenic reductase is responsible for enzymatic reduction of As(V) [6,7]. The mobility of As in soil depends on the biological and physico-chemical properties of the environment. Important factors that affect As mobility mainly include the presence of other ions in the environment, pH, Eh (oxidation–reduction potential), soil aeration, temperature, the proportion of clays and organic material, and the activity of rhizosphere microbial communities [8]. The speciation of As in the plant–soil system is also dynamic and depends on several soil factors, as well as the plant species [9,10].

Roots are usually the first tissue exposed to As, where the metalloid inhibits root extension and proliferation. Upon translocation to the shoots, As can severely inhibit plant growth by slowing or arresting expansion and biomass accumulation, as well as compromising plant reproductive capacity through losses in fertility, yield, and fruit production [11]. Although arsenic is not redox-active, it can stimulate the generation of reactive oxygen species through the conversion of arsenate to arsenite, and can thus induce lipid peroxidation and cellular damage [2]. The disturbance of plant mineral nutrition is the main cause of yield decrease, the most frequent sign of As toxicity [12]. The consequence of disturbance of mineral homeostasis is the darkening of roots and the drying of leaves, which indicates the inhibited transport of water into tissues [3,12,13]. The plant reacts to reduced water content in tissues in different ways. In conditions of short-term water stress, plants try to regulate water content by reducing stomatal openings and also the rate of transpiration. However, under conditions of long-term water deficit, there are often changes in the density and/or size of stomata [14]. Stomata also have a strong influence on the characteristics associated with photosynthesis. Many studies have indicated that As can inhibit the flow of photosynthetic electrons, destroy antennae pigments, down-regulate PSII proteins, and inhibit the water-splitting complex of the oxidizing site of PSII. Damage to chloroplast structure during treatment with high arsenic levels imply functional changes to integrated photosynthetic processes [15].

One of the many interesting paradoxes related to As toxicity is that plant growth is stimulated at low As concentrations [11,16]. The stimulatory effect of low doses and toxicity of higher doses of chemicals on plant growth is referred to as hormesis, which comes into consideration mainly in cases of toxic non-essential, often trace, elements [17]. In a certain range of concentrations of these elements, tolerance to the given element is shown, or a stimulatory effect of the element is observed [17,18]. Low doses of arsenic (AsIII or AsV), for example, stimulated the growth of maize, tomato, onion, rye and wheat [5,19,20,21,22,23]. The hormetic effect has also been observed in cases of Li, Cu, Cd, Pb, Al, Cr, Se and Sb [24,25,26,27,28,29]. The stimulatory effect is shown especially in tolerant species (cultivars), and depends on many factors such as the pH of the environment, the deficiency or toxicity of another ion in the growth medium, the element’s duration of action, its availability to plants, and different levels of the element’s translocation from roots to shoots [30,31,32]. In addition, in order to effectively adapt to changed environmental conditions, the plant creates certain compromises, which often results in a non-synchronous change in morphological, physiological and biochemical parameters. Some parameters do not change, and some even worsen compared to the control [33,34]. It is also important to note that the hormetic response of plants is often examined in hydroponic systems, in which the degree of toxicity of risk elements is different from that in natural conditions. The results of these studies are therefore more difficult to apply in practice.

Maximal stimulation at low doses tends to be mild, and is usually less than twice the control response [35]. Low-dose hormetic stimulation is an adaptive response that reflects an environmentally induced altered phenotype, and provides a quantitative estimate of biological plasticity [18,36].

Although knowledge about hormesis mechanisms is growing, the phenomenon has still not been sufficiently explained. It has been explored in several studies that tolerance to metal stress, plant growth, biomass, photosynthetic pigments, and gas exchange characteristics can be increased by the application of endogenous and exogenous amendments of ABA (abscisic acid) or SA (salicylic acid) [24,28,37,38]. Stimulation tends to increase plant defense, mainly due to the induction of synthesis of defense molecules (stress proteins), secondary metabolites, and the alteration of antioxidant enzyme activity and the reduction of oxidative stress by inhibiting the lipid peroxidation of membranes [24]. The activities of SOD (superoxide dismutase), APX (ascorbate peroxidase), GPX (guaiacol peroxidase), and CAT (catalase) play important roles in maintaining ROS (reactive oxygen species) in lower doses and duration of As exposure [39]. On the other hand, it has been shown that several hormesis mechanisms are triggered by the induction of oxidative stress [40,41]. Nitrogen oxide (NO) molecules most probably also play an important role in this process [15,42]. The stimulatory effect of arsenic may also be due to its chemical similarity to phosphorus. Both elements form oxyanions that compete for adsorption sites on positively charged soil particles [43,44]. Attempts are currently being made to use the hormetic stimulation of plant parameters to increase resilience to multiple stressors.

The main objectives of this study were (1) to evaluate the effect of As(III) on the growth and selected physiological characteristics of shoots of two maize genotypes (cvs. Chapalu and MvNK 333), and (2) to evaluate the differences in the hormetic response of the tested cultivars to As.

## 2. Results

### 2.1. Effect of Arsenic on Growth and Relative Water Content

**Figure 1:**
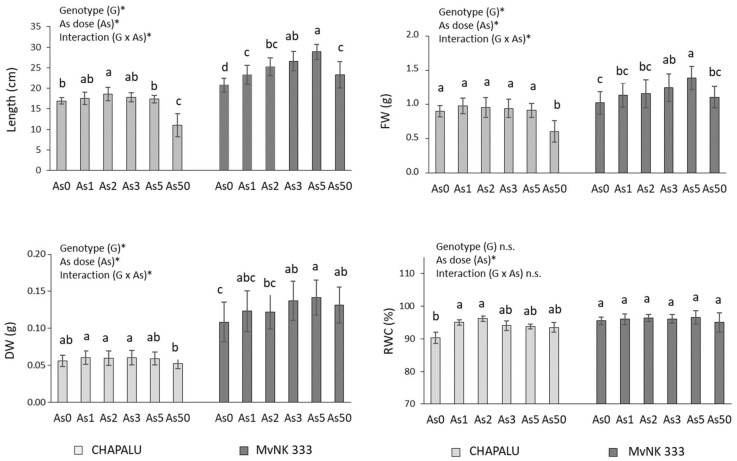
Effect of arsenic (As) on length, fresh weight (FW), dry weight (DW) and relative water content (RWC) of shoots of two maize cultivars and their statistical evaluation. Data are presented as means ± SD from three biological replicates (n = 45 for FW and DW, n = 6 for RWC). As0, As1, As2, As3, As5 and As50—arsenic addition to soil in mg kg^−1^. The differences between variants of the experiment were determined using the Kruskal–Wallis test followed by the Dunn’s post hoc test for multiple comparisons. Different letters on the top of bars indicate a significant difference (*p* < 0.05) between variants of the experiment for each cultivar. Data were also examined using two-way ANOVA with the factors As dose (As), genotype (G), and interaction (G x As). *—significance at 0.05 level; n.s.—not significant.

In the pot experiment, the test doses of arsenic stimulated the growth of shoots of cv. Chapalu by 1.77% (As1), 6.30% (As2), 4.18% (As3), and 3.53% (As5) (length of shoots), only the change at the dose of As2 compared to As0 was statistically significant. As a result of the As50 dose, shoot length decreased by 35.25% (Figure 1). The biomass of this cultivar did not change significantly in the range of As0–As5 (Figure 1). As a result of As50, FW decreased by 32.32% and DW by 7.14%. A stronger stimulation of growth was observed in the case of cultivar MvNK 333. The dose of As1–5 mg kg^−1^ increased the shoot length (by 12.90–43.34%) and the biomass of the shoots (FW by 10.68–35.92%, DW by 23.85% and 32.11%) (Figure 1). We also observed a weaker stimulation at a dose of As50 (increase in length by 12.20%, FW by 8.82%, and DW by 18.18%).

The tolerance indexes (TI) determined on the basis of growth parameters were above 100% for all test doses of As (except for As50 in the case of cv. Chapalu) in both cultivars. Cultivar MvNK 333 was generally more tolerant than cv. Chapalu (Table 1, Figure 1).

We also observed a weak insignificant increase of 0.27–1.03% in RWC in the case of cv. MvNK 333, and a higher increase of 0.95–3.64% in the case of cv. Chapalu with significant stimulation at doses As1 and As2 (Figure 1).

### 2.2. Effect of Arsenic on the Content of Photosynthetic Pigments

**Figure 2:**
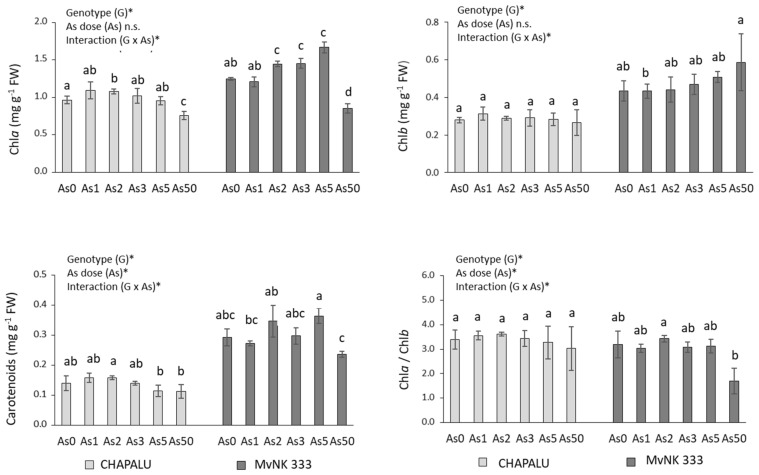
Effect of arsenic on photosynthetic parameters of two cultivars of maize and their statistical evaluation. Data are presented as means ± SD from three biological replicates (n = 6). As0, As1, As2, As3, As5 and As50—arsenic addition to soil in mg kg^−1^. The differences between variants of the experiment were determined using the Kruskal–Wallis test followed by Dunn’s post hoc test for multiple comparisons. Different letters on the top of bars indicate a significant difference (*p* < 0.05) between variants of the experiment for each cultivar. Data were also examined using two-way ANOVA with the factors As dose (As), genotype (G), and interaction (G x As). *—significance at 0.05 level; n.s.—not significant.

In the case of cv. Chapalu, arsenic caused a significant increase in the Chl*a* at a dose of As2 and minimal changes in other parameters of photosynthesis in the dose range As0–As5 (Figure 2). The dose of As50 reduced the Chl*a* content compared to As0 by 21.45%, and the As5 and As50 doses reduced the carotenoid content by 14.29% and 21.43%.

In the case of cultivar MvNK 333, more significant changes were observed at Chl*a* in the dose range As2–As5 (increase by 15.96, 16.76 and 33.76%) and carotenoids at the dose of As5 (increase by 24.13%). As a result of As50, there was a decrease in the content of Chl*a* (by 31.4%), and an insignificant decrease in the content of carotenoids (by 17.25%) and the ratio of Chl*a*/Chl*b* (by 46.71%) (Figure 2).

### 2.3. Effect of Arsenic on Stomatal Density and Stomata Size

**Figure 3:**
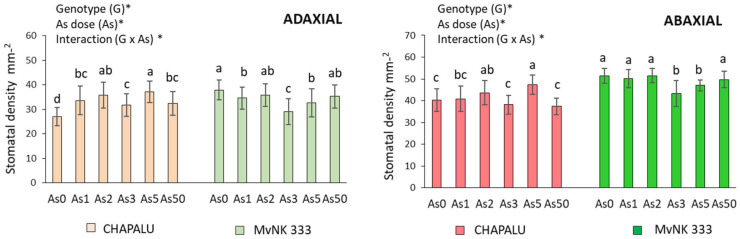
Effect of arsenic on stomatal density in adaxial and abaxial side of leaves of two cultivars of maize. Data are presented as means ± SD from three biological replicates (n = 45). As0, As1, As2, As3 and As50—arsenic addition to soil in mg kg^−1^. The differences between variants of the experiment were determined using the Kruskal–Wallis test followed by Dunn’s post hoc test for multiple comparisons. Different letters on the top of bars indicate a significant difference (*p* < 0.05) between variants of the experiment for each cultivar. Data were also examined using two-way ANOVA with the factors As dose (As), genotype (G), and interaction (G x As). *—significance at 0.05 level.

The tested cultivars of maize reacted to As differently as far as the number of stomata on leaves is concerned. While in the case of cv. Chapalu the increasing dose of As caused an increase in the number of stomata on the adaxial side of leaves (by 25.45–42.51%, with the maximum at As5), and it caused a decrease in the number of stomata (by 5.47–23.43%) in the case of cultivar MvNK 333 (Figure 3). We observed a similar trend on the abaxial side of leaves (Figure 3).

**Figure 4:**
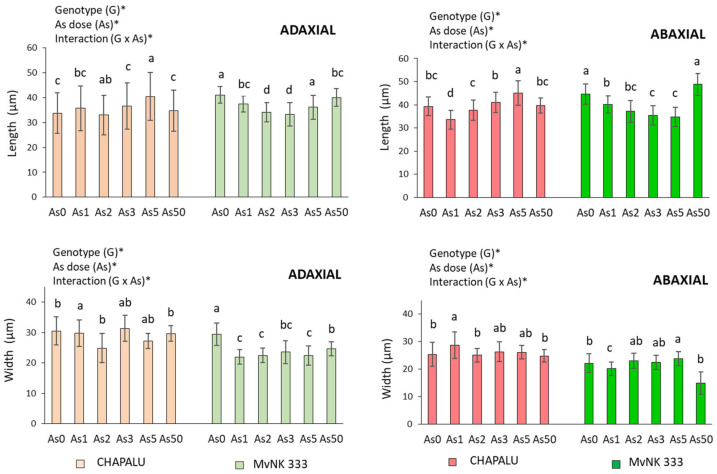
Effect of arsenic on size of stomata in adaxial and abaxial side of leaves of two cultivars of maize and their statistical evaluation. Data are presented as means ± SD from three biological replicates (n = 45). As0, As1, As2, As3, As5 and As50–arsenic addition to soil in mg kg^−1^. The differences between variants of the experiment were determined using the Kruskal–Wallis test followed by Dunn’s post hoc test for multiple comparisons. Different letters on the top of bars indicate a significant difference (*p* < 0.05) between variants of the experiment for each cultivar. Data were also examined using two-way ANOVA with the factors As dose (As), genotype (G), and interaction (G x As). *—significance at 0.05 level.

Only the dose of As5 had a stimulating effect on the length of stomata in the case of cv. Chapalu (increase by 19.82% on the adaxial side and by 14.81% on the abaxial side compared to As0) (Figure 4).

**Figure 5:**
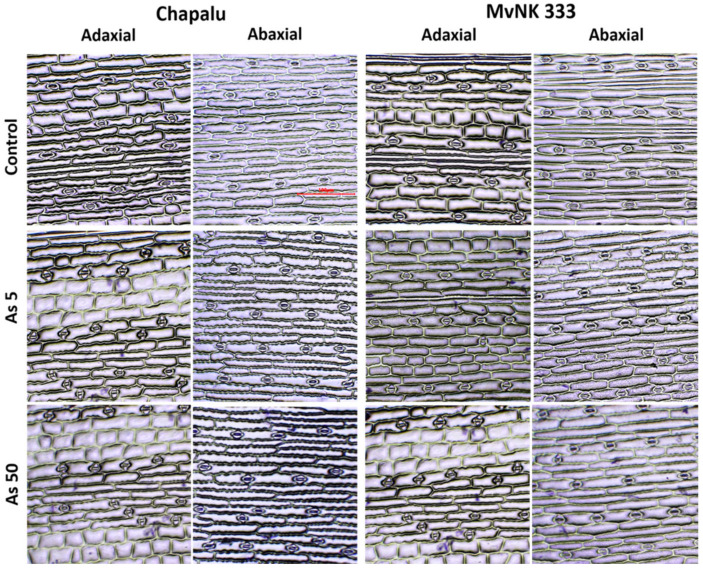
Light microscope images of stomata of maize (cvs. Chapalu and MvNK 333) treated with distilled water (control) and arsenic (As addition: 5 and 50 mg kg^−1^ of soil). Scale bar = 100 µm.

In the case of cv. MvNK 333, stomata were shortened with increasing doses of As (As1–As5) (by 2.94, 16.98, 19.10 and 12.08% on the adaxial side, and by 10.38, 17.06, 20.75 and 22.366% on the abaxial side). The length of the stomata in this cultivar reached the level of As0 at a dose of As50 (Figure 4). We noted a direct narrowing of stomata at all doses of As (by 16.08–25.25%) on the adaxial side of the leaf of cv. MvNK 333. On the abaxial side of the leaf of this cultivar, the changes in stomata were dose-dependent (significant narrowing at As1 and As50 by 9.10 and 32.44%, expansion at dose of As5 by 7.47%). In the case of cv. Chapalu, there was a narrowing of stomata on the adaxial side of the leaf due to As2 and As5 (by 2.33 and 10.86%). On the abaxial side of the leaf, the changes were minimal, and a significant expansion of stomata was caused by As1 (by 13.18%) (Figure 4). No stomata abnormalities were observed, even at the highest arsenic doses (Figure 5).

### 2.4. Arsenic Accumulation in Shoots

**Figure 6:**
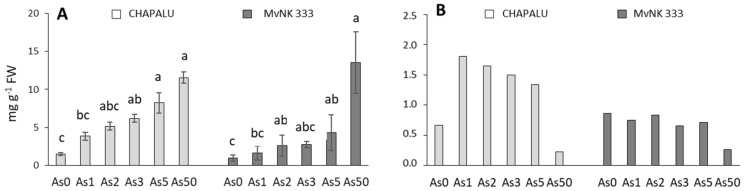
Arsenic (As) content in shoots (**A**) of two cultivar of maize (cvs. Chapalu and MvNK 333) and bioaccumulation factor (**B**). The differences between variants of the experiment were determined using the Kruskal–Wallis test followed by the Dunn’s post hoc test for multiple comparisons (*p* < 0.05) (**A**). Different letters on the top of bars indicate a significant difference (*p* < 0.05) between variants of the experiment for each cultivar. Data are presented as means ± SD from three biological replicates (n = 6). As0, As1, As2, As3, As5 and As50—arsenic addition to soil in mg kg^−1^.

With increasing dose of As in the soil, there was an increased accumulation of As in the shoots of both maize cultivars. The Chapalu cultivar accumulated higher amounts of arsenic (1.5, 3.86, 5.16, 6.20, 8.23 and 11.54 mg kg^−1^ DW) in the shoots than the MvNK 333 cultivar (0.97, 1.65, 2.63, 2.74, 4.34 and 13.54 mg kg^−1^ DW) in the dose range As0–As50 (Figure 6). At a dose of As50, both cultivars accumulated approximately the same amount of As (Figure 6).

The difference in the accumulation potential of the shoots of the two cultivars of corn is also reflected in the bioaccumulation factor (BAF) values (Figure 6B), which also point to changes in As accumulation with respect to As content in the soil. The highest value of BAF (1.81) was determined at a dose of As1 in the case of cv. Chapalu; with increasing dose of As, BAF values gradually decreased. In the case of cv. MvNK 333, the BAF did not differ significantly in the dose range of As0–As5, and at a dose of As50, the BAF decreased to approximately the same level as in the case of cv. Chapalu (Figure 6B).

### 2.5. Evaluation of the Effect of Genotype and Arsenic Dose on Tested Parameters

The effect of genotype and arsenic dose on tested parameters is also confirmed by the results of two-way ANOVA. All tested parameters (except Chl*b* and RWC) were dependent on arsenic dose and genotype. Changes in Chl*b* values were determined by a dose of As as well as the interaction of As and genotype; RWC was also determined with respect to dose of As. We observed the interaction of As and genotype for most of the tested parameters (Figure 1, Figure 2, Figure 3 and Figure 4).

**Figure 7:**
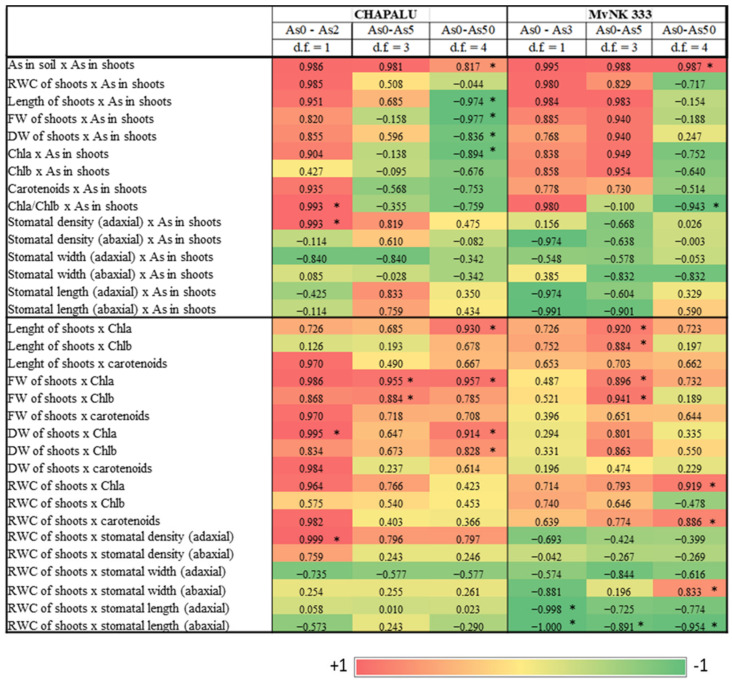
Correlation analysis in a heat map for three level of As dose. *—significant correlation at *p* < 0.05; d.f.—degrees of freedom.

To more clearly evaluate the hormetic manifestation of As in the observed corn cultivars, correlations were defined between the parameters in three ranges/levels of As dose: As0–As2, As0–As5 and As0–As50 (Figure 7). We observed a strong positive correlation in both cultivars of corn between the As content in soil and the As content in shoots (R = 0.817–0.995), as well as between the As content in shoots and growth parameters (length of shoots, biomass of shoots) (Figure 7). We also noted a positive relationship between As content and photosynthetic parameters, but in a different range of As doses in two cultivars (Figure 7). The strongest correlation was recorded in the range of doses where the highest stimulating effect of As was recorded (for cv. Chapalu in the range As0–As2, and for cv. MvNK 333 in the dose range As0–As5). The correlation matrix for the As0–As50 range also indicates that, at the As50 dose, a turning point occurred when the positive correlation relations for several parameters change to negative or from negative to positive. These conclusions are also supported by the data presented in Figure 1, Figure 2, Figure 3 and Figure 4. On the other hand, for several stomatal parameters, we noted a negative relationship with RWC, as well as with As content in shoots. It is interesting to note that the number of stomata and their length in the dose range As0–As5 (with a maximum at As5) showed a positive relationship in the case of the Chapalu cultivar, but a negative relationship in the case of cv. MvNK 333 (Figure 7). The positive relationship between growth and photosynthetic parameters was manifested across the entire range of As doses (Figure 7).

## 3. Discussion

The toxicity of pollutants on higher plants can be readily evaluated in terms of those pollutants’ impact on early-stage root elongation and seedling growth. The stimulation of shoot growth as an effect of As (As1–As5) in both tested cultivars suggests that they are tolerant cultivars, which is also confirmed by the values of TI (100% and higher). The cv. MvNK 333 showed a generally higher tolerance (TI > 100% even at a dose of As50), which may be related to a lower content of accumulated As in tissues (Figure 6). The results of analysis indicated that the hormetic effect of As (stimulation by a lower dose and inhibition by a higher dose) was more pronounced on the growth and content of photosynthetic pigments in both cultivars. The strong positive correlation between As content and growth parameters was strongest in the As1–As2 dose range in the case of cv. Chapalu and in the As2–As3 dose range in the case of the cv. MvNK 333 (Figure 1, Figure 2, Figure 3, Figure 4 and Figure 7). Stimulation of maize growth was also observed in tolerant cultivars Dongdan90 as an effect of 2 mg L^−1^ As [45]. In contrast, however, doses of 2 and 5 mg L^−1^ resulted in decreased growth, leaf area, biomass, chlorophyll, carotenoid, and protein content in maize leaves after five days of As treatment [23]. In the study conducted by Ci et al. [46], concentrations of 12.5 and 25 mg kg^−1^ in the soil promoted maize growth and the nutritional quality of the grain, whereas higher concentrations (50 and 100 mg As kg^−1^ of soil) presented toxic effects for the crop. The assumption that hormesis is more common in tolerant cultivars was also confirmed in our experiments.

The stimulatory effect of As was also manifested in the content of Chl*a* and carotenoids in the leaves of cv. NvNK 333, but was less expressed in the case of cv. Chapalu (Figure 2). The inhibitory effect of the highest dose was manifested in both cultivars by a decrease in the content of Chl*a*, carotenoids and the Chl*a*/Chl*b* ratio (Figure 2). An increased accumulation of photosynthetic pigments due to lower AsIII (1- 2 mg L^−1^) doses was also recorded in the leaves of wheat [47] and onion [20], with contrasting decreased accumulation in maize leaves. Increased photosynthetic pigments have also been reported in maize leaves exposed to lower doses of cadmium and lead [28]. In shoots, hormesis is often associated with enhanced photosynthesis activity, which improves plant development [33,48]. Modulation of the proportion between chlorophylls *a* and *b* (Chl *a*/*b* ratio) can improve photosynthesis efficiency in plants that face environmental challenges, such as low N availability, excess light, and metal (metalloid) exposure [33,48]. Increased concentration in carotenoids could act as part of the protection mechanism against As-induced oxidative stress [12,49]. This mechanism was probably most effective at As doses, when the strongest stimulation was recorded in individual cultivars (Figure 7). Correlation analyses confirmed the positive relationship between pigment content and biomass content in both cultivars (Figure 7), but also highlighted that changes depend on genotype as well as As dose (Figure 7). Different manifestations of hormesis depending on photosynthetic pigment type have also been reported in other plants [33]. An inverted U-shaped dose–response relationship of chlorophylls and photosynthesis response to stresses was found to be consistent with the biomass response pattern of higher terrestrial plants. However, the underlying mechanisms of photosynthesis stimulation by environmental pollutants in terms of hormesis remain underexplored [33].

In the next part of the experiment, we focused on stomatal characteristics. This is because the modification of stomata frequency and sizes as a response to environmental stress is an important way in which plants control the absorption of pollutants and regulate water status in tissues [50,51]. The change in the size of the stomata is related to the process of their opening and closing, and the retention of water in the tissues. The importance of stomata in the regulation of water content in the tissues of the tested maize cultivars is also supported by the results of our analyzes (strong positive correlation between As content in tissues and RWC, and predominantly negative correlation between stomata size and RWC due to lower As doses). Correlation analyses also suggested that water regulation mechanisms are a little different in the two cultivars. While cv. Chapalu tended to increase the number of stomata on both sides of leaves, cv. MVNK 333 instead responded to the given conditions by reducing the size of stomata (Figure 3 and Figure 7). An increase in the number of stomata due to metal stress was also observed by other authors in less tolerant plant cultivars [52,53,54]. According to Melo et al. [55], the reduction in transpiration surface under water deficit conditions is compensated by increased stomatal density and reduced stomatal size. A decrease in the number of stomata and decreased leaf transpiration rate also constitute an effective plant resistance mechanism against drought [56]. Shortening the stomata on the adaxial side of leaves of *Arachis hypogaea* L. after the application of cadmium is also reported by [57] in their study. Leaf RWC gives a strong indication of a plant’s response to different environmental conditions [50]. An increase in RWC (albeit insignificant) was also recorded in sunflower at As doses of 20 and 40 mg kg^−1^ of soil [58]. In xerohalophyte plant species *Atriplex atacamensis* after 14 days of As treatment (100 µM), root and leaf water content did not change, but stem water content increased [59]. A significant decrease in leaf RWC was observed in As-treated soybean compared to control plants after one day of treatment, mainly with As(III), but was re-established after four and eight days. These results indicate that the decrease in total water content (WC) at the beginning of stress reaction acts as a signal for induction of mechanisms leading to the regulation of water content in tissues [14]. The long-term adaptation of poplar callus to Cd was also described by Labancová et al. [60]. Increased RWC is probably related to increased transpiration, which has also been observed with the effects of other hormetic agents [61]. Increased transpiration under the given conditions may indicate increased nutrient absorption efficiency with the potential for greater nutrient supply to aboveground tissues [33]. This mechanism is probably more likely to be applied in cv. Chapalu, and may also be related to the increased amount of As in the tissues. The phenomenon was also observed in *Pteris vittata* [62].

There is probably no general mechanism for stomatal response to the presence of risk elements in the environment [63]. Several authors have reported that with an increased concentration of heavy metals, the number of epidermal stomata increased [56]. Yet other authors have noted the decrease in stomata number due to Cd [64,65]. Variability in the number of stomata on the upper and lower sides of leaves has also been reported in maize leaves depending on genotype as well as the type of heavy metals [66]. These contradictions can also be caused by the differing reactions of single parts of leaves to different stress types [14,54].

Total As content in the shoots increased with increasing dose of As in the soil in both cultivars, and was generally higher in the Chapalu cultivar (Figure 6). Higher accumulation of As in shoots of this cultivar could be the cause of an earlier hormetic response, with respect to the shift of the hormetic curve peak compared to the MvNK 333 cultivar. While the effect of the highest dose of As dose (As50) began to show symptoms of toxicity in the Chapalu cultivar (decrease in shoot length and fresh weigth of shoots), the MvNK 333 cultivar tolerated the dose better. This shift is also recorded in the weaker correlations between As content in shoots and tested parameters in the case of the Chapalu cultivar in the dose range As0–As5 (Figure 7). The different accumulation of As in the tested maize cultivars may also be related to their differing ability to absorb water and transport it to shoots [62]. The influence of genotype on RWC was also confirmed by two-way ANOVA (Figure 1). The differences in accumulation potential of tested corn varieties are also reflected in the values of bioaccumulation factor (BAF) (Figure 6B). BAF, based on total concentrations of As in soils and plant tissues, is commonly used to assess the potential of As uptake from soils [67]. The relatively high values of BAF in both cultivars indicate the efficient transport of As into shoots. However, similar BAF values were recorded at much higher doses of As (>100 mg kg^−1^ soil) [9]. According to the statutory limits of As concentration in cereals and food crops in different countries [68,69,70,71] (Table 2), the cultivation of cv. MvNK 333 in soils contaminated with As may represent a certain threat to the food chain, as the given variety is recommended for silage and green matter consumption. We do not expect the As content limits in the grains of these varieties to be exceeded, given the low amounts measured so far at much higher doses of As in the soil [9,72].

On the other hand, the high tolerance of these varieties to As (As1–As5, in the case of cv. MvNK 333, as well as As50), as well as the accumulation of a relatively high content of As in shoots, indicates the possibility of using these varieties in the process of the phytostabilization or phytoremediation of soils contaminated with arsenic.

In general, we recorded stimulation of growth, increase in RWC, and increase in content of Chl*a* and carotenoids in the tested cultivars of maize. The given changes were dependent on genotype and range of As doses. The stimulation maximum for cv. Chapalu was represented by doses of As1 and As2, in the case of cv. MvNK 333 by doses As3 and As5, while in this variety, a stimulating effect cannot be excluded, even at higher doses of As. The results also indicate that BAF values are more important than the actual amount of accumulated As in the tissues in terms of estimating the maximum stimulation. Although hormetic manifestations closely related to changes in the number and size of stomata were observed, we did not observe a typical hormetic response of stomata in the given range of As doses. That the hormetic display often manifests itself in some parameters but not others, and moreover in another dose range the acting agent, is—we conclude—largely determined by genotype (current response to a given dose of metal, or accumulation potential) and different sensitivities of individual physiological processes to a certain metal dose. The stimulation of parameters/functions against the background of suppression of others is probably a manifestation of biological plasticity, the aim of which is to adapt to changed environmental conditions. The unsynchronized stimulation of plant parameters exposed to low doses of stress has also been observed by other authors [36,73].

## 4. Materials and Methods

### 4.1. Plant Material and Growth Condition

Two varieties of corn *Zea mays* L. (cultivars Chapalu and MvNK 333) were used for the experiment. The selected corn varieties are hybrids with high tolerance to drought, with high productivity and low post-harvest grain moisture. Both varieties are also adaptable to different soil-ecological conditions. The Chapalu variety is a hybrid with lower height, and does not produce too much biomass. It concentrates its energy on the creation of fully developed cobs of corn. It is grown for grain. Cultivar MVNK 333 is a hybrid of corn with intense growth. This hybrid is also recommended for silage and green matter consumption.

In the pot experiments, plastic containers (diameter 15 cm) were filled with 300 g dry peat substrate (Klasmann KTS 2, *Klasmann*-Deilmann GmbH, Germany, pH KCl. 6.7, gravimetric soil water content max. 70%, EC v µS 400, total arsenic (As) content 1.13 mg kg^−1^, water soluble arsenic (As) content 0.15 mg kg^−1^ soil, nitrogen (N) content 1.65 g kg^−1^, carboneum (C organic) content 81.59 g kg^−1^, phosphorus content (P) 0.38 g kg^−1^, potassium (K) content 0.43 g kg^−1^), to which 20 seeds of corn were sown. After sowing, arsenic in doses of 1, 2, 3, 5 and 50 mg kg^−1^ of soil (As1 to As5) was applied to the soil substrate. The given doses were applied once at the beginning of the experiment in a volume of 150 mL of aqueous solution. In several studies, the water-soluble As level in the soil was better correlated with the As content in the plant than the total As content in the soil [5,67,74]. We therefore related all analyses to the content of water-soluble As in soil. Therefore, the amounts of As in the soil were 0.15, 1.15, 2.15, 3.15, 5.15, and 50.15 mg kg^−1^ soil. The amounts applied are given as variants As0 (control), As1, As2, As3, As5 and As50. The solutions of arsenic (AsIII) were prepared from certified reference material for arsenic (Sigma-Aldrich, Darmstadt, Germany). The plants were then regularly watered (every third day), with the water volume corresponding to maximum 80% of the soil’s water-holding capacity (150 mL). The pot experiment was conducted in a climate box in order to provide constant conditions for the experiment (temperature 23 °C, humidity 60–70%; illumination periods of 12 h light/12 h dark, radiation intensity of 400 µmol m^−2^ s^−1^). The experiment was set up in three replicates. After 12 days of growth in contaminated soil, the following parameters were measured: shoot length, FW and DW of shoots, photosynthetic pigments content, stomata number, and stomatal size. The plants were analyzed at the three-leaf stage (V3) in accordance with Ritchie et al. [75]. Parameters such as RWC, pigment content, and stomatal characteristics were examined in two plants of each variant and biological replicate.

### 4.2. Determination of Growth Parameters and Tolerance Index

The fifteen equally developed plants of each variant and repetition (total 45/genotype) were used to evaluate shoot length and biomass. Shoot length was measured with a ruler. After determining the fresh weight (FW) of shoots, they were dried in an incubator for 48 h at 60 °C to constant weight and then weighed using analytical scales. The tolerance index (TI) was calculated as a ratio of the mean dry weight (DW) of plants grown in the presence of arsenic, and the mean DW of the control plants expressed as a percentage [76].

### 4.3. Determination of Photosynthetic Pigments

The contents of photosynthetic pigments: chlorophyll *a* (Chl*a*), chlorophyll *b* (Chl*b*), and carotenoids were determined spectrophotometrically (spectrophotometer UV-2601, Shimadzu, Japan) in fully developed third assimilating leaves at three wavelengths: 470 nm, 648 nm, and 664 nm. Acetone (80%) was used for pigment extraction. The amount of pigments (chlorophylls and carotenoids) was obtained using the following formulas [77]:

Chl*a* = 13.36A_664_ − 5.19A_648_

For chlorophyll *a* content

Chl*b* = 27.43A_648_ − 8.12A_664_

For chlorophyll *b* content

Carotenoids (x + c) = 1000A_470_ − 2.13 Chl*a* − 97.64 Chl*b*/209

For total carotenoid content.

Pigments were determined in six replicates in each variant of the experiment.

### 4.4. Determination of Relative Water Content (RWC)

Leaf material was weighed to determine the fresh weight (FW), and placed in distilled water at +4 °C for 12 h; thereafter, turgid weight (TW) was recorded. Finally, the samples were dried in an oven at 70 °C for 48 h and dry weights (DW) were recorded. RWC (in %) was obtained using the formula: RWC = (FW ࢤ DW)/(TW ࢤ DW) × 100 Dhopte and Manuel [78]. RWC was determined in six replicates for each variant of the experiment and genotype.

### 4.5. Determination of Number and Size of Stomata

The number and size of stomata were assessed after 12 days of growth in contaminated soil on upper (adaxial) and lower (abaxial) sides of leaves using clear nail polish, tape, and a glass slide in accordance with Xu and Zhou [79]. Imprints were taken from the middle portion of the blade between the midrib and the leaf margin, on the third leaf from three plants per treatment of the experiment. Leaves of similar size and maturity were used. The stomatal samples were collected in conditions with a temperature of 25 °C between 9:30 and 11:00 a.m. In total, 24 microscopic fields of each epidermis and variant of experiments were randomly selected and examined using the optical microscope (Zeiss Axioplan, Oberkochen, Germany) and then counted. The number of stomata was expressed per mm^2^ of leaf area. To determine stomatal guard cell length (stoma length) and guard cell pair width (stoma width), 45 randomly chosen stomata on each leaf (variant of experiments) were measured at 400× magnification. Images were obtained using a Sony DXCS500 digital camera and analyzed with AxioVision AC software (Zeiss, Jena, Germany).

### 4.6. Determination of As in Shoots and Soil

Dried and ground shoots (0.5 g ± 0.1 mg) and soil (1 g ± 0.1 mg) were used to determine total arsenic content. The material was decomposed in 10 mL of ultrapure nitric acid, with the addition of 2 mL of ultrapure hydrogen peroxide (Analytika, Prague) at high temperature and pressure in a SW-4 microwave decomposer (Berghof, Bavaria, Germany). After decomposition, the samples were quantitatively transferred to vials and made up to a final volume of 20 mL with water. The determination of arsenic concentration was performed on an ICP MS/MS 8800 instrument (Agilent Technologies, Tokyo, Japan). First, the approximate arsenic content was determined by the “quick scan” method of one random sample using one calibration standard of 10 µg L^−1^ due to the appropriate setting of the calibration curve range. Based on the obtained information, the calibration dependence of eight points was set in the range of 0–10 µg L^−1^. A stock solution of arsenic (Analytika, Prague) with a concentration of 1.000 g L^−1^ was used to prepare the standards. The reaction oxygen mode was used to measure As, which ensured the good removal of possible interferences. A certified reference material (CRM AN-BM01, Strawberry leaves for shoots and CRM Metranal 33 for soil) was analyzed to ensure the accuracy of the ICP-MS procedure). Ultrapure water (Merck-Millipore, France) was used to prepare all the necessary standards and solutions, and all solutions, reagents, and samples were stored in plastic containers. Limits of detection (LOD) and limits of quantification (LOQ) for As were: 0.017 mg kg^−1^ and 0.025 mg kg^−1^.

Water-soluble arsenic in soil was determined according to the standard ČSN EN 12457-4 [80]. Soil (1 g ± 0.1 mg) and Milli-Q water were mixed in 1:10 ratio, and the mixed solution was shaken for 24 h using a rotary shaker. Arsenic in the filtered solution was measured on an ICP MS/MS 8800 instrument (Agilent Technologies, Japan). The determination (calibration and analysis) was verified by two reference materials with satisfactory yield: SLRs-6 (River Water Reference Material), and 1640 (SRM—Trace Elements in Natural Water).

### 4.7. Determination of Bioacumulation Factor for As

Shoot bioaccumulation factor (BAF) was defined as the ratio of total As concentrations in the plant material to total As concentrations in the soil [67].

### 4.8. Statistical Analysis

The obtained results were statistically analyzed using XLSTAT software. Basic statistical characteristics (arithmetic mean, standard deviation) were determined. The differences between variants of the experiment were examined using the Kruskal–Wallis test, followed by Dunn’s post hoc test *(p <* 0.05). Two-way analysis of variance (ANOVA, *p <* 0.05) was performed to determine the effects of plant genotype, arsenic dose, and their interaction on the tested parameters. The heat map correlation analysis (Pearson) was estimated to show the statistical relationship between the quantitative parameters.

## 5. Conclusions

The effects of low doses of arsenic on tested cultivars of maize cultivated in contaminated soil can be regarded as beneficial. Both tested cultivars showed a high level of tolerance to test doses of As (TI_As1–As5_ of 100% and higher), with cultivar MvNK 333 showing generally higher tolerance. The influence of the highest dose (As50) showed a predominantly inhibitory effect of As in both varieties with a more pronounced impact on growth and the content of photosynthetic pigments. We did not observe a typical stomatal hormetic response in the given range of As doses. An increase in shoot length, biomass, carotenoids, and chlorophylls may represent defence mechanisms to growing under arsenic-impacted conditions. Our data show that leaf epidermal cell adjustments are flexible components of plant defenses even at low metal doses, and possibly help to compromise the structural and functional needs of plant (tissue) under arsenic stress. The results also suggested that the hormetic effect itself is cultivar-dependent, with different cultivars having varying abilities to transport, accumulate, and tolerate these elements in their tissues. Since the hormetic effect of elements is often influenced by the monitored element’s duration of action, the evaluation of a given aspect could contribute to a deeper analysis of the issue. Knowing hormesis mechanisms and also identifying plant cultivars tolerant to As should be considered an important strategy in enhancing plants’ tolerance to As and in the remediation of As contaminated soils.

## Figures and Tables

**Table 1:** Tolerance indexes (TI) in % determined on the basis of growth parameters of shoots.

			Chapalu					MvNK 333		
TI (%)	As1	As2	As3	As5	As50	As1	As2	As3	As5	As50
Length of shoots	103.59	109.70	104.82	102.66	64.75	112.28	121.64	128.19	139.19	112.20
Fresh weight of shoots	108.83	106.55	104.91	101.78	67.52	111.08	113.08	121.73	135.52	108.06
Dry weight of shoots	107.91	106.35	107.63	105.54	93.69	113.54	112.51	126.19	130.45	118.18

**Table 2:** Regulation limits of As concentration in cereals or food crops established by different countries.

Country	Regulation Item	Statutory Limit	Reference
Switzerland	fodder	4 mg kg^−1^ DW	[5]
Canada	food crops	1 mg kg^−1^ FW	[68]
China	rice	0.15 mg kg^−1^ DW	[69]
Australia	cereals	1 mg kg^−1^ FW	[70]
Germany	cerals	1 mg kg^−1^ FW	[71]
India	cerals	1 mg kg^−1^ FW	[71]
The Netherlands	cereals	1 mg kg^−1^ FW	[71]

DW—dry weight, FW—fresh weight.

## Data Availability

The data presented in this study are available on request from the corresponding author.
